# Prospective multicenter study of camrelizumab in real-world settings for asian patients with esophageal squamous cell carcinoma

**DOI:** 10.1186/s12885-024-13196-4

**Published:** 2024-11-18

**Authors:** Tingting Li, Yaqing Dai, Xiaobin Fu, Qunrong Cai, Dongmei Ke, Qiwei Yao, Jiancheng Li

**Affiliations:** 1 Clinical Oncology School of Fujian Medical University, Fujian Cancer Hospital, 420 Fuma Road, Jin’an District, Fuzhou, Fujian 350014 China; 2 Department of Radiation Oncology, The Second Affiliated Hospital of Fujian Medical University, Quanzhou, Fujian 362000 China; 3 Department of Radiation Oncology, The First Affiliated Hospital of Xiamen University, Teaching Hospital of Fujian Medical University, Xiamen, 361003 China

**Keywords:** Esophageal squamous cell carcinoma, Camrelizumab, Lung immune prognostic index, Real-world study

## Abstract

**Background:**

In this study, we aimed to evaluate the real-world efficacy and safety of camrelizumab and identify clinicolaboratory factors that predict treatment outcomes in patients with unresectable advanced, recurrent, or metastatic esophageal squamous cell carcinoma (ESCC) receiving camrelizumab.

**Methods:**

Herein, 174 patients with unresectable advanced, recurrent, or metastatic ESCC treated with camrelizumab monotherapy (*n* = 30), camrelizumab + chemotherapy (CT; *n* = 91), and camrelizumab + radiotherapy (RT; *n* = 53) between October 1, 2019 and October 1, 2022 were included.

**Results:**

The median follow-up time was 20 months (range, 1–34 months). The median progression-free survival (PFS) and overall survival (OS) of the whole cohort were 8 months [95% confidence interval (CI), 6.5–9.5 months] and 14 months (95% CI, 11.2–16.8 months), respectively. After multivariate analysis, receiving > 4 cycles of camrelizumab was identified as an independent predictor of better PFS [hazard ratio (HR), 0.56; 95% CI, 0.38–0.827; *P* = 0.004] and OS (HR, 0.532; 95% CI, 0.341–0.83; *P* = 0.005). An intermediate-to-poor lung immune prognostic index (LIPI) was identified as an independent predictor of worse PFS (HR, 1.505; 95% CI, 1.032–2.196; *P* = 0.034) and OS (HR, 1.657; 95% CI, 1.094–2.51; *P* = 0.017). The disease control rate of patients in the camrelizumab monotherapy group, camrelizumab + CT group, and camrelizumab + RT group was 92.3% (95% CI, 74.9–99.1%), 90.6% (95% CI, 82.3–95.9%), and 96.1% (95% CI, 86.8–99.5%), respectively. The treatment-related adverse events (AEs) of grade 3 or higher were reported in 67 patients (38.5%). The most common treatment-related AEs were decreased neutrophil count (23.0%), decreased white blood cell count (19.5%), anemia (7.5%), and pneumonitis (4.6%). One patient (0.6%) died from a treatment-related AE of immune checkpoint inhibitor-induced myocarditis.

**Conclusion:**

Camrelizumab was safe and effective as both monotherapy and part of a combination therapy. Longer PFS and OS were associated with receiving > 4 cycles of camrelizumab and having a good LIPI. LIPI can be used as a prognostic biomarker for ESCC patients receiving camrelizumab + RT.

**Trial registration:**

ClinicalTrial.gov Identifier: CHICTR2000039499. Registered: 19th October 2020.

## Background

According to the International Agency for Research on Cancer (IACR), esophageal carcinoma (EC) ranks seventh among most common cancers (604,000 new cases) and sixth for most cancer-related deaths (544,000 death cases) worldwide in 2020 [1]. Squamous cell carcinoma and adenocarcinoma are the two main histologic subtypes of esophageal cancer. Esophageal squamous cell carcinoma (ESCC) accounts for approximately 90% of cases in parts of Asia and Africa. In addition, esophageal adenocarcinoma (EAC) represents nearly 70% cases in high-income countries like the United States and Europe [2, 3]. Before 2019, the practice guidelines of the National Comprehensive Cancer Network recommended a combination of fluoropyrimidine and platinum-based chemotherapy as the first-line therapy for unresectable advanced and metastatic ESCC cases, which could achieve an objective response rate (ORR) of 33% and a median overall survival (OS) of 8–10 months. In the event of first-line therapy failure, the subsequent second-line treatment options are limited. Paclitaxel, docetaxel, or irinotecan have showed anti-tumor efficacy for ESCC and have all been clinically available as second-line chemotherapy over the past decades; however, they showed dismal disease control and survival benefit of a median OS of approximately 7 months [4–7]. Therefore, new systemic treatment alternatives are urgently required to improve the prognosis of unresectable advanced and metastatic EC patients.

Recently, accumulating studies have shown that anti-programmed death receptor 1 (anti-PD-1) inhibitor shows promising efficacy with tolerable adverse effects as a first- and second-line therapy in multiple solid tumors, including advanced and metastatic EC [8–10]. Nivolumab and pembrolizumab significantly prolonged survival in recurrent, locally advanced, or metastatic ESCC with a PD-L1 combined positive score (CPS) of 10 or higher in the second-line settings. Subsequently, a huge breakthrough also occurred after the adoption of PD-1 inhibitors in combination with chemotherapy in recurrent, locally advanced, or metastatic ESCC regardless of the PD-L1 expression. Therefore, both nivolumab and pembrolizumab have been approved by the United States Food and Drug Administration as both first- and second-line therapy in recurrent, locally advanced, or metastatic ESCC [11–13]. Given the high incidence of ESCC in China and the enormous financial burden of the cost of nivolumab and pembrolizumab, there is an unmet medical need to develop cost-effective PD-1 inhibitors for patients in China [14]. Camrelizumab (Jiangsu Hengrui Pharmaceuticals Co, Ltd), a fully humanized, selective IgG4-k monoclonal antibody against PD-1, also showed potent antitumor activity with manageable toxicity in ESCC [15]. ESCORT was a randomized double-blind, phase 3 study of second-line camrelizumab versus chemotherapy (docetaxel or irinotecan) in patients with advanced or metastatic ESCC. Camrelizumab was superior to chemotherapy in terms of the median overall survival in the total population (8.3 months vs. 6.2 months, *P* = 0.001). The incidences of serious treatment-related adverse events were similar in the camrelizumab group and chemotherapy group (16% vs. 15%) [16]. The phase 3 ESCORT-1st study of first-line camrelizumab + chemotherapy versus placebo + chemotherapy in advanced or metastatic ESCC showed a better overall survival with the former than with the latter (median overall survival, 15.3 months vs. 12 months, *P* = 0.01), and the incidences of grade 3 or higher treatment-related adverse events were similar between the two groups (63.4% vs. 67.7%, respectively) [17].

Based on data from ESCORT and ESCORT-1st studies in patients with unresectable advanced or metastatic ESCC, camrelizumab monotherapy and camrelizumab + chemotherapy were recommended as second- and first-line therapies by the China National Medical Products Administration in this population, respectively. However, for a wide variety of patients with uncontrolled unresectable advanced, recurrent, or metastatic ESCC, real-world data on the use and outcomes of different treatment patterns in routine clinical practice are currently lacking. In addition, outcome benefits of camrelizumab may be limited to a special portion of patients. Therefore, the primary aim of this multicenter, observational, prospective, real-world study was to assess the safety and efficacy of camrelizumab combination therapy and monotherapy as first- and second-line therapies for ESCC patients. The secondary aim was to identify the potential clinicolaboratory factors that predict the treatment outcomes in ESCC patients receiving camrelizumab in a real-world setting.

## Methods

### Research design and eligibility criteria

Given that camrelizumab was approved as second-line therapy in June 2020 and first-line therapy in December 2021 by the China National Medical Products Administration, only a minority of unresectable advanced, recurrent, or metastatic ESCC patients had received camrelizumab in clinical practice before January 2022. In addition, the safety and efficacy of camrelizumab combination therapy and monotherapy as first- and second-line therapies for ESCC patients in the real-world setting remain unclear. Therefore, a real-world, multicenter, prospective, observational study including Chinese patients was designed to fill this gap in knowledge. Therefore, in this study, we recruited consecutive patients with unresectable advanced, recurrent, or metastatic ESCC who had been treated with camrelizumab combination therapy or monotherapy in the Department of Oncology or Department of Radiation Oncology in 20 hospitals in China between October 1, 2019 and October 1, 2022. Cancer stage was determined based on the 8th American Joint Committee on Cancer TNM staging system. The inclusion criteria were as follows: age > 18 years, histologically confirmed ESCC, ≥ 1 measurable lesion according to the Response Evaluation Criteria in Solid Tumors (RECIST) version 1.1, Eastern Cooperative Oncology Group performance status (ECOG PS) score 0 or 1, good medical compliance, adequate organ function, and basically normal findings for routine blood test, biochemical investigation, and blood clotting function test. The exclusion criteria were as follows: a history of or having an active autoimmune disease, a history of or having congenital or acquired immunodeficient disease, a history of ICI treatment, a history of interstitial lung disease, having active hepatitis B or C viral infection, ongoing pregnancy or lactation, having drug-induced pneumonia, active symptomatic pneumonia, or radiation-induced pneumonia with steroid treatment, and having undergone previous therapies involving antibiotics, steroids, or other immunosuppressants within 2 weeks before the study treatment. Ultimately, 178 registered consecutive patients with unresectable advanced, recurrent, or metastatic ESCC were initially enrolled. Four patients (2.2%) dropped out, and 174 eligible patients were included in the study. Among them, 12 patients did not consent to undergo radiographic examination after the treatment. Thus, the remaining 162 patients were assessed for short-term efficacy. This study was approved by the ethics committee of the Fujian Cancer Hospital. Written informed consent was sought and obtained from each patient according to recommendations of the Declaration of Helsinki.

## Treatments

### Immunotherapy

A median of 4 (1–20) cycles of camrelizumab were administered in all 174 patients with unresectable advanced, recurrent, or metastatic ESCC. Camrelizumab was intravenously given over 30 min at a dose of 200 mg every 3 weeks (Q3W) as systemic immunotherapy until disease progression, complete response, patients’ withdrawal of consent, or unacceptable toxicity.

### Chemotherapy

A total of 119 (68.4%) patients were treated with two or more cycles of chemotherapy. Systemic chemotherapy comprised fluoropyrimidine, irinotecan, cisplatin, oxaliplatin, nedaplatin, S1, capecitabine, docetaxel, and taxane. Chemotherapy was continued until disease progression, completion of 4–6 cycles of chemotherapy, patients’ withdrawal of consent, or unacceptable toxicity.

### Radiotherapy

A total of 53 (30.5%) patients received intensity-modulated radiotherapy (IMRT). Among these, 36 patients were prescribed a radical dose of 50–63 Gy in 25–30 fractions (5 fractions per week) for the primary gross tumor volume, gross volume of the involved lymph nodes, and/or clinical tumor volume. In addition, 17 patients were prescribed a palliative dose of 10–45 Gy in 5–15 fractions (5 fractions per week) for gross tumor volume of the distant metastases. Radiotherapy continued until disease progression, patients’ withdrawal of consent, unacceptable toxicity, or completion of total dose of radiotherapy.

## Definition of lung immune prognostic index

Peripheral blood tests were performed before initiating camrelizumab treatment, and data on absolute neutrophil count (ANC), white blood cell count (WBC), and lactate dehydrogenase (LDH) levels were collected. The lung immune prognostic index (LIPI) was defined based on the combination of the derived neutrophil-to-lymphocyte ratio (dNLR) and the LDH level. The dNLR was defined as ANC divided by WBC minus ANC. LIPI scores were defined on the basis of the following cutoff values: dNLR > 3 and LDH > 250 (our institution’s upper limit of normal) were defined as a score of 2; dNLR > 3 and LDH ≤ 250 *or* dNLR ≤ 3 and LDH > 250 were defined as a score of 1; and dNLR ≤ 3 and LDH ≤ 250 were defined as a score of 0. In this study, LIPI scores of 1 and 2 were merged to represent the intermediate-to-poor LIPI group, and a LIPI score of 0 represented the good LIPI group.

## Follow up, assessments, and outcomes

Among the 174 eligible patients, 14 patients’ survival status could not be assessed as they could not be telephonically reached (loss to follow-up rate: 8.0%). Thus, the last survival time of these patients who were lost to follow-up was considered as the censoring date. Tumor response was independently assessed every 6 weeks by two investigators using radiographic examination according to the RECIST version 1.1. The main radiographic examination modalities used in this study was magnetic resonance imaging and computed tomography (CT) scanning. After treatment discontinuation, patients were telephonically followed up every 2 months to evaluate their survival status until death. Adverse events were evaluated and recorded continuously for up to 30 days after the end of treatment discontinuation (90 days for serious adverse events) and assessed according to the Common Terminology Criteria for Adverse Events, version 4.03.

The primary clinical endpoints in all patients were overall survival (OS) and progression-free survival (PFS). OS was defined as the interval between the date camrelizumab initiation and death from any cause or last follow-up. PFS was defined as the interval between the date of camrelizumab initiation and disease progression. October 2022 was the last censoring date for evaluating primary clinical endpoints. The secondary clinical endpoints in all patients were objective response rate (ORR), disease control rate (DCR), safety, and tolerability. ORR was defined as the proportion of patients with complete response (CR) or partial response (PR). DCR was defined as the proportion of patients with CR, PR, and stable disease (SD).

### Statistical analysis

All recorded data were analyzed using SPSS (version 23.0; IBM Corp, Armonk, NY, USA), GraphPad Prism (version 9.3.0, GraphPad Software, San Diego, California, USA), SAS (version 9.4, SAS Institute Inc), and R software (version 4.0.5, The R Foundation for Statistical Computing). The camrelizumab cycle was converted into classification variables using the median cycles of camrelizumab. The OS and PFS curves were drawn using the Kaplan–Meier method, and the log-rank test was used to analyze the differences in 2-year OS and PFS between the groups. The clinical characteristics used in univariable analysis were sex, age, ECOG PS, clinical stage, treatment type, line of immunotherapy, drinking history, LIPI, and camrelizumab cycles. Cox proportional hazards regression analysis was further performed in the clinical characteristics to identify the independent factors influencing the 2-year OS of patients with unresectable advanced, recurrent, or metastatic ESCC. Differences between the intermediate-to-poor LIPI group and good LIPI group were compared using the Chi-square test or Fisher’s exact test. ORR and DCR were presented with accompanying 95% CIs calculated on the basis of the Clopper–Pearson exact method, and the comparisons of ORR and DCR among camrelizumab monotherapy, camrelizumab + CT, and camrelizumab + RT were made using the stratified Cochran–Mantel–Haenszel test. Hazard ratios and 95% confidence intervals (CIs) were estimated for each factor. All tests were two-sided, and a P value of < 0.05 was considered statistically significant.

## Results

### Baseline demographics and characteristics

Table 1 summarizes the baseline characteristics of the study patients. In the entire cohort of 174 patients, the median patient age was 63 years (range, 31–86 years), the ratio of male (*n* = 136) to female (*n* = 38) was 3.58:1, and the median follow-up duration was 20 months (range, 1–34 months).

**Table 1:** Characteristics of 174 patients with unresectable advanced, recurrent, or metastatic esophageal squamous cell carcinoma (ESCC)

Characteristics	N (%)
**Sex**	
Male	136 (78.2)
Female	38 (21.8)
**Age**,** median (range)**	63 (31–86) years
**Clinical stage**	
II	3 (1.7)
III	16 (9.2)
IV	155 (89.1)
**Cancer presentation**	
Local advanced	19 (10.9)
Primary metastatic disease	38 (21.8)
Recurrent disease	117 (67.2)
**Treatment type**	
Camrelizumab monotherapy	30 (17.2)
Camrelizumab + CT	91 (52.3)
Camrelizumab + RT	53 (30.5)
**Line of immunotherapy**	
First line	88 (50.6)
Second line	86 (49.4)
**ECOG**	
0	11 (6.3)
1	163 (93.7)
**Smoking history**	
No	108 (62.0)
≤ 20 yeas	29 (16.7)
> 20 years	37 (21.3)
**Drinking history**	
No	137 (78.7)
Yes	37 (21.3)
**Tumor location**	
Cervical	9 (5.2)
Upper	23 (13.2)
Middle	112 (64.4)
Lower	26 (14.9)
NA	4 (2.3)
**LIPI**	
0	109 (62.6)
1	59 (33.9)
2	6 (3.5)
**Camrelizumab cycles**	
≤ 4	103 (59.2)
> 4	71 (40.8)

Abbreviations: CT = chemotherapy, RT = radiotherapy, ECOG = Eastern Cooperative Oncology Group, LIPI = lung immune prognostic index

### Clinical outcomes of the whole cohort

For the entire cohort, the median PFS and median OS durations of the whole cohort were 8 months [95% CI, 6.5–9.5 months] and 14 months (95% CI, 11.2–16.8 months), respectively. The 12- and 24-month PFS rate was 36.3% and 22.1%, respectively. The 12- and 24-month OS rate was 53.4% and 31.2%, respectively.

**Fig. 1:**
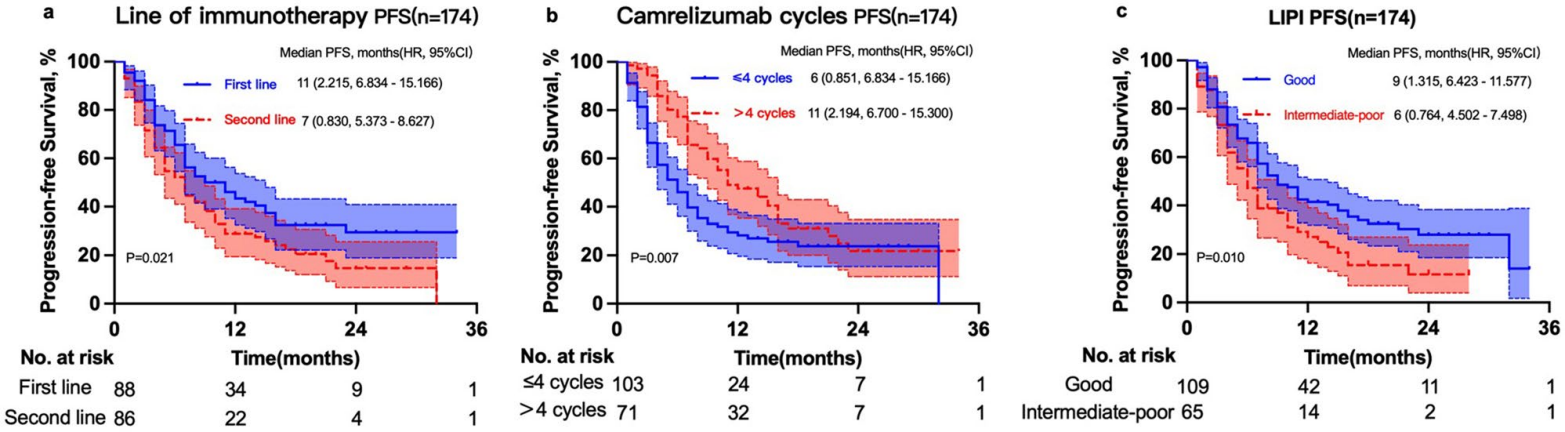
Progression-free survival of 174 patients with unresectable advanced, recurrent, or metastatic esophageal squamous cell carcinoma (ESCC) in groups receiving camrelizumab as first-line therapy and second-line therapy (**a**); groups receiving ≤ 4 cycles and > 4 cycles of camrelizumab (**b**); and groups with good lung immune prognostic index (LIPI) and intermediate-to-poor LIPI (**c**)

Progression events occurred in 70.1% (122/174) patients in the whole cohort. The median PFS was 7 months (95% CI: 5.4–8.6 months) in patients who received camrelizumab as the second-line therapy versus 11 months (95% CI: 6.8–15.2 months) in patients who received camrelizumab as the first-line therapy (χ2 = 5.338, *P* = 0.021). The 12- and 24-month PFS rates were 29.0% and 14.7% in patients who received camrelizumab as the second-line therapy, respectively, and 46.1% and 29.5% in patients who received camrelizumab as the first-line therapy, respectively (Fig. 1a). The median PFS was 6 months (95% CI: 4.3–7.7 months) in patients who received ≤ 4 cycles of camrelizumab versus 11 months (95% CI: 6.7–15.3 months) in patients who received > 4 cycles of camrelizumab (χ2 = 7.242, *P* = 0.007). The 12- and 24-month PFS rates were 28.3% and 23.8% in patients who received ≤ 4 cycles of camrelizumab, respectively, and 47.5% and 21.8% in patients who received > 4 cycles of camrelizumab, respectively (Fig. 1b). The median PFS was 6 months (95% CI: 4.5–7.5 months) in the intermediate-to-poor LIPI group versus 9 months (95% CI: 6.4–11.6 months) in the good LIPI group (χ2 = 6.591, *P* = 0.01). The 12- and 24-month PFS rates were 27.1% and 11.6% in the intermediate-to-poor LIPI group, respectively, and 41.5% and 28.1% in the good LIPI group, respectively (Fig. 1c).

**Fig. 2:**
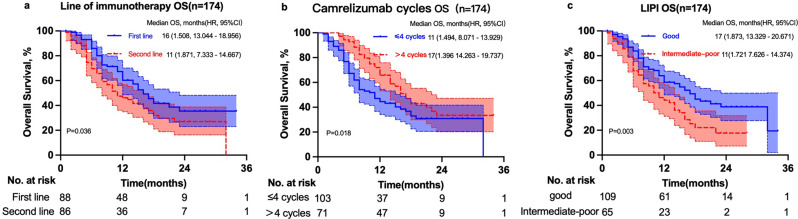
Overall survival of 174 patients with unresectable advanced, recurrent, or metastatic ESCC in groups receiving camrelizumab as first-line therapy and second-line therapy (**a**); groups receiving ≤ 4 cycles and > 4 cycles of camrelizumab (**b**); and groups with good LIPI and intermediate-to-poor LIPI (**c**)

Death occurred in 56.9% patients (99/174) in the whole cohort. The median OS was 11 months (95% CI: 7.3–14.7 months) in patients who received camrelizumab as the second-line therapy versus 16 months (95% CI: 13.0–15.2 months) in patients who received camrelizumab as the first-line therapy (χ2 = 4.408, *P* = 0.036). The 12- and 24-month OS rates were 46.2% and 27.0% in patients who received camrelizumab as the second-line therapy, respectively, and 60.3% and 35.5% in patients who received camrelizumab as the first-line therapy, respectively (Fig. 2a). The median OS was 11 months (95% CI: 8.1–13.9 months) in patients who received ≤ 4 cycles of camrelizumab versus 17 months (95% CI: 14.3–19.7 months) in patients who received > 4 cycles of camrelizumab (χ2 = 5.637, *P* = 0.018). The 12- and 24-month OS rates were 44.5% and 30.9% in patients who received ≤ 4 cycles of camrelizumab, respectively, and 65.8% and 33.4% in patients who received > 4 cycles of camrelizumab, respectively (Fig. 2b). The median OS was 11 months (95% CI: 7.6–14.4 months) in the intermediate-to-poor LIPI group versus 17 months (95% CI: 13.3–20.7 months) in the good LIPI group (χ2 = 8.624, *P* = 0.03). The 12- and 24-month PSF rates were 44.3% and 17.7% in the intermediate-to-poor LIPI group, respectively, and 64.0% and 38.9% in the good LIPI group, respectively (Fig. 2c).

Univariate analysis showed that receiving camrelizumab as the first-line therapy (χ2 = 5.338; *P* = 0.021), receiving > 4 cycles of camrelizumab (χ2 = 7.242; *P* = 0.007), and having a good LIPI (χ2 = 6.591; *P* = 0.01) were positively associated with prolonged PFS. In addition, receiving camrelizumab as the first-line therapy (χ2 = 4.408; *P* = 0.036), receiving > 4 cycles of camrelizumab (χ2 = 5.637; *P* = 0.018), and having a good LIPI (χ2 = 8.624; *P* = 0.003) were positively associated with prolonged OS (Tables 2 and 3). After multivariate analysis, receiving > 4 cycles of camrelizumab was identified as an independent predictor of better PFS (HR, 0.56; 95% CI, 0.38–0.827; *P* = 0.004) and OS (HR, 0.532; 95% CI, 0.341–0.83; *P* = 0.005). An intermediate-to-poor LIPI was identified as an independent predictor of worse PFS (HR, 1.505; 95% CI, 1.032–2.196; *P* = 0.034) and OS (HR, 1.657; 95% CI, 1.094–2.51; *P* = 0.017).

**Table 2:** Univariate analysis of 2-year progression-free survival and overall survival in 174 patients with unresectable advanced, recurrent, or metastatic ESCC

	PFS					OS		
	n	MST (month)	χ²	*P* value		MST (month)	χ²	*P* value
**Sex**			0.410	0.522			0.093	0.761
Male	136	7				14		
Female	38	11				15		
**Age**			0.475	0.491			0.017	0.896
≤ 70	144	7				14		
> 70	30	15				16		
**ECOG**			0.460	0.498			1.262	0.261
0	11	23				23		
1	163	8				14		
**Clinical stage**			0.014	0.906			0.033	0.855
II–III	19	15				15		
IV	155	7				14		
**Treatment type**			5.792	0.055			4.685	0.096
Camrelizumab monotherapy	30	5				10		
Camrelizumab + CT	91	9				17		
Camrelizumab + RT	53	9				14		
**Line of immunotherapy**			5.338	0.021			4.408	0.036
First line	88	11				16		
Second line	86	7				11		
**Drinking history**			0.093	0.760			0.990	0.320
No	137	8				14		
Yes	37	7				17		
**LIPI**			6.591	0.010			8.624	0.003
Good	109	9				17		
Intermediate–poor	65	6				11		
**Camrelizumab cycles**			7.242	0.007			5.637	0.018
≤ 4	103	6				11		
> 4	71	11				17		

**Table 3:** Multivariate analysis of 2-year progression-free survival and overall survival in 174 patients with unresectable advanced, recurrent, or metastatic ESCC

	PFS					OS		
	n	HR	95% CI	*P* value		HR	95% CI	*P* value
**Sex**				0.711				0.718
Male	136	1				1		
Female	38	0.912	0.559–1.487			0.904	0.522–1.566	
**Age**				0.401				0.995
≤ 70	144	1				1		
> 70	30	0.801	0.478–1.344			1.002	0.572–1.756	
**ECOG**				0.444				0.141
0	11	1				1		
1	163	1.388	0.599–3.217			2.165	0.774–6.052	
**Clinical stage**				0.904				0.893
II–III	19	1				1		
IV	155	0.963	0.521–1.780			0.954	0.447–1.906	
**Treatment type**				0.071				0.070
Camrelizumab monotherapy	30	1				1		
Camrelizumab + CT	91	0.559	0.340–0-920	0.022		0.516	0.294–0.907	0.021
Camrelizumab + RT	53	0.622	0.358–1.080	0.092		0.635	0.345–1.170	0.146
**Line of immunotherapy**				0.114				
First line	88	1				1		0.233
Second line	86	1.364	0.928–2.006			1.302	0.844–2.008	
**Drinking history**				0.321				0.065
No	137	1				1		
Yes	37	0.783	0.483–1.270			1.699	0.968–2.984	
**LIPI**				0.034				0.017
Good	109	1				1		
Intermediate–poor	65	1.505	1.032–2.196			1.657	1.094–2.510	
**Camrelizumab cycles**				0.004				0.005
≤ 4	103	1				1		
>4	71	0.560	0.380–0.827			0.532	0.341–0.830	

### Clinical outcomes of different treatment patterns

**Fig. 3:**
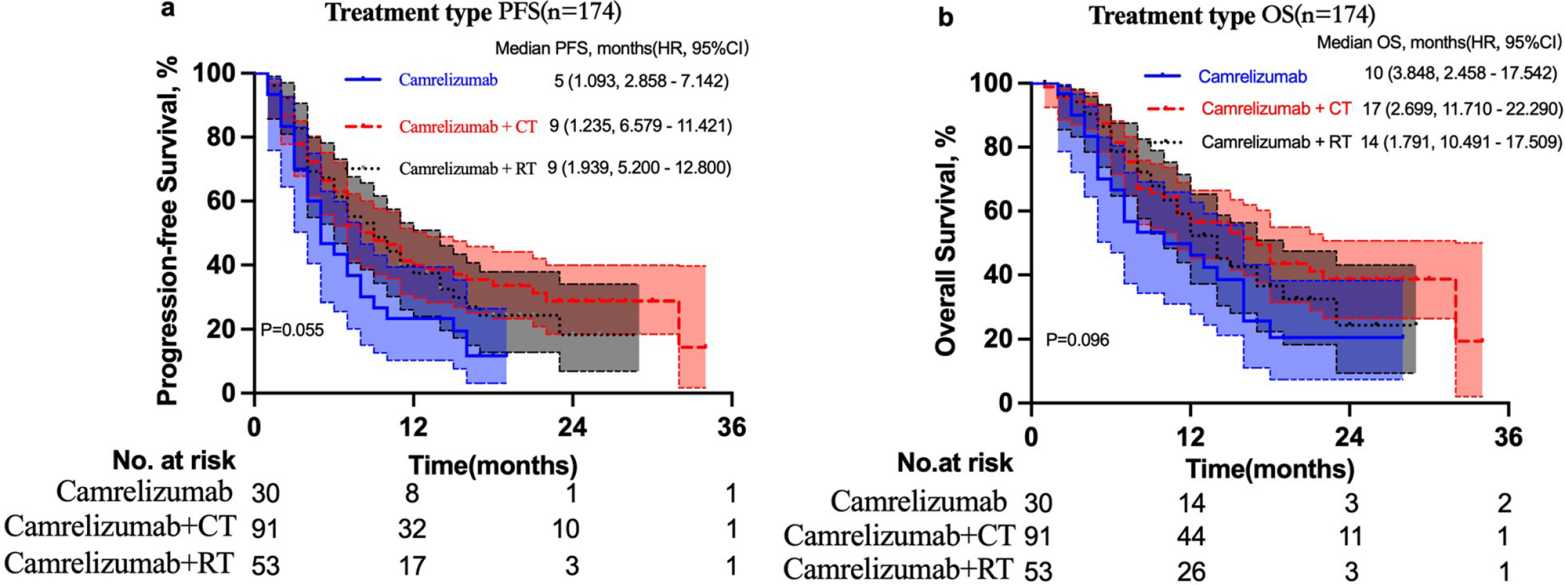
Progression-free survival (**a**) and overall survival (**b**) of patients with unresectable advanced, recurrent, or metastatic esophageal squamous cell carcinoma ESCC in camrelizumab monotherapy, camrelizumab + CT, and camrelizumab + RT groups

To investigate the impact of different treatment methods on patients’ prognoses, we subdivided all patients into three groups according to whether they combined with chemotherapy or radiotherapy or neither. The camrelizumab monotherapy group included 17.2% (30/174) patients who received only camrelizumab. The camrelizumab + chemotherapy (CT) group included 52.3% (91/173) patients who received camrelizumab combined with chemotherapy but not radiotherapy. The camrelizumab + radiotherapy (RT) group included 30.5% (53/174) patients who received camrelizumab + RT but with or without chemotherapy. Among the patients in the camrelizumab + RT group, 28 patients received chemotherapy and 25 did not receive chemotherapy. In camrelizumab monotherapy, camrelizumab + CT, and camrelizumab + RT groups, the median PFS rates were 5 months (95% CI, 2.8–7.1 months), 9 months (95% CI, 6.6–11.4 months), and 9 months (95% CI, 5.2–12.8 months), respectively (*P* = 0.055). The Kaplan–Meier estimates for 12-month PFS rates of the three groups were 23.3%, 40.0%, and 35.6%, respectively, and for 24-month PFS rates were 11.7%, 28.8%, and 22.1%, respectively. Camrelizumab + CT led to a significantly prolonged PFS than camrelizumab monotherapy (χ2 = 5.489; *P* = 0.019). Moreover, camrelizumab + RT led to a prolonged PFS than camrelizumab monotherapy (χ2 = 3.16; *P* = 0.075); however, the difference was not statistically significant (Fig. 3a).

For patients receiving camrelizumab monotherapy, camrelizumab + CT, and camrelizumab + RT, the median OS was 10 months (95% CI, 2.5–17.5 months), 17 months (95% CI, 11.7–22.3 months), and 14 months (95% CI,11.7–22.3 months), respectively (*P* = 0.096). The Kaplan–Meier estimates for 12-month OS rates for the three treatment groups were 37.5%, 56.5%, and 46.2%, respectively, and for 24-months OS rates were 18.3%, 38.7%, and 20.5%, respectively. Camrelizumab + CT led to a significantly prolonged OS than camrelizumab monotherapy (χ2 = 4.576; *P* = 0.032). Conversely, the OS did not significantly differ between camrelizumab + RT and camrelizumab monotherapy groups (χ2 = 1.454; *P* = 0.228; Fig. 3b).

**Fig. 4:**
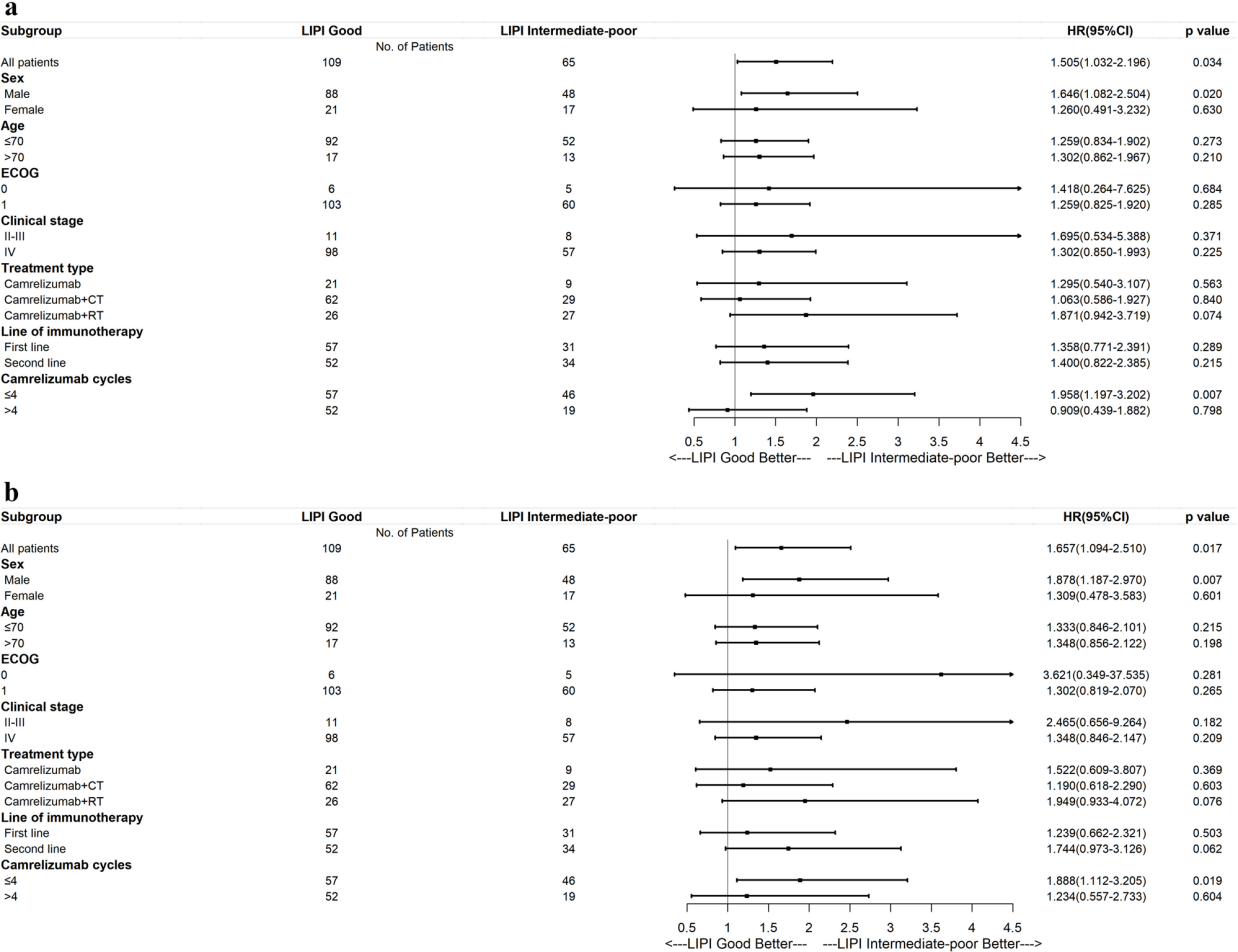
Forest plots for subgroup analysis of the (**a**) progression-free survival and (**b**) overall survival of patients with unresectable advanced, recurrent, or metastatic ESCC according to the LIPI groups in camrelizumab + RT, camrelizumab monotherapy, and camrelizumab + CT

A PFS and OS benefit with LIPI was consistently observed across the three different treatment methods and other variables, such as sex, age, ECOG PS, clinical stage, line of immunotherapy, and camrelizumab cycles. Baseline characteristics were well balanced in the two groups (Table 4). In the subgroup analyses of PFS, male sex (HR, 1.646; 95% CI, 1.082–2.504; *P* = 0.02) and patients with camrelizumab ≤ 4 (HR, 1.958; 95% CI, 1.197–3.202; *P* = 0.007) were found to be associated with significantly prolonged PFS in the intermediate-to-poor LIPI group (Fig. 4a). In the subgroup analyses of OS, male sex (HR, 1.878; 95% CI, 1.187–2.97; *P* = 0.007) and patients with camrelizumab ≤ 4 (HR, 1.888; 95% CI, 1.112–3.126; *P* = 0.019) were found to be associated with significantly prolonged OS in the intermediate-to-poor LIPI group. In addition, in patients who received camrelizumab + RT, we also found the PFS (HR, 1.871; 95% CI, 0.942–3.719; *P* = 0.074) and OS (HR, 1.949; 95% CI, 0.933–4.072; *P* = 0.076) to be slightly better in patients with intermediate-to-poor LIPI than in those with good LIPI; however, the difference was not statistically significant (Fig. 4b).

**Table 4:** Characteristics of the 174 patients with unresectable advanced, recurrent, or metastatic ESCC with good lung immune prognostic index (LIPI) and intermediate-to-poor LIPI

Characteristics	LIPI		χ²	*P* value
	Good *N* (%)	Intermediate-poor *N* (%)		
**Sex**			1.132	0.287
Male	88	48		
Female	21	17		
**Age**			0.553	0.457
≤ 70 years	92	52		
> 70 years	17	13		
**ECOG**			-	0.749^a^
0	6	5		
1	103	60		
**Clinical stage**			0.206	0.650
II-III	11	8		
IV	98	57		
**Line of immunotherapy**			0.345	0.557
First line	57	31		
Second line	52	34		
**Smoking status**			0.045	0.832
No	67	41		
Yes	42	24		
**Drinking history**			0.204	0.652
No	87	50		
Yes	22	15		

If N ≥ 40 and 1 ≤ theoretical frequency (T) < 5, the Fisher exact test was used to compared the influencing factors

### Short-term efficacy outcomes of the whole cohort

Table 5 summarizes the objective tumor response to camrelizumab. In the 162 patients with measurable disease, CR, PR, and SD were achieved in 5 (3.1%), 66 (40.7%), and 79 (45.4%) patients, respectively. The ORR and DCR were 43.8% (95% CI, 36.1–51.8%) and 92.6% (95% CI, 87.4–96.1%), respectively. The CR, PR, and SD rates in the three groups were as follows: camrelizumab monotherapy group [2 (7.7%), 10 (38.5%), and 12 (46.2%), respectively], camrelizumab + CT group [2 (2.4%), 38 (44.7%), and 37 (43.5%), respectively], and camrelizumab + RT group [1 (2.0%), 18 (35.3%), and 30 (58.8%), respectively]. The ORR and DCR rates in the three groups were as follows — camrelizumab monotherapy group: 46.2% (95% CI, 26.6–66.6%) and 92.3% (95% CI, 74.9–99.1%), respectively; camrelizumab + CT group: 47.1% (95% CI, 36.1–58.2%) and 90.6% (95% CI, 82.3–95.9%), respectively; and camrelizumab + RT group: 37.3% (95% CI, 24.1–51.9%) and 96.1% (95% CI, 86.8–99.5%), respectively (Table 5). No significant difference in ORR (*P* = 0.521) or DCR (*P* = 0.498) was observed among the three different treatment methods.

**Table 5:** Best responses to camrelizumab monotherapy, camrelizumab + CT, and camrelizumab + RT

	ALL	Camrelizumab monotherapy	Camrelizumab + CT	Camrelizumab + RT	*P* value
Response assessment CR, n	5	2	2	1	-
Response assessment PR, n	66	10	38	18	-
Response assessment SD, n	79	12	37	30	-
Response assessment PD, n	12	2	8	2	-
Drop out, n	12	4	6	2	-
Total, n	174	30	91	53	-
ORR, n (%; 95% CI)	71 (43.8; 36.1–51.8)	12 (46.2; 26.6–66.6)	40 (47.1; 36.1–58.2)	19 (37.3; 24.1–51.9)	0.519
DCR, n (%; 95% CI)	150 (92.6; 87.4–96.1)	24 (92.3; 74.9–99.1)	77 (90.6; 82.3–95.9)	49 (96.1; 86.8–99.5)	0.496

### Safety and feasibility

During the camrelizumab treatment period, treatment-related adverse events (AEs) occurred in 167/174 (96.0%) patients (Table 6). Treatment-related pneumonitis (any grade) was reported in 22 (12.6%) patients. Immune-related reactive capillary endothelial proliferation (any grade) was reported in 94 (54.0%) patients. Treatment-related AEs of grade 3 or higher were reported in 67 (38.5%) patients, with the most common ones being decreased neutrophil count (23.0%), decreased WBC count (19.5%), anemia (7.5%), and pneumonitis (4.6%). Treatment-related AEs led to treatment interruption of any treatment component in 16 patients (9.2%), with treatment interruption being the most prominent in patients with pneumonitis (4.6%). Patients’ decision and disease progression led to treatment interruption of any treatment component in 13 (7.5%) and 46 (26.4%) patients. One patient (0.6%) died from a treatment-related AE of immune checkpoint inhibitor-induced myocarditis (Table 6).

**Table 6:** Summary of treatment-related adverse events

	All patients (%)	Grade 1–2 (%)	Grade ≥ 3 (%)
Pneumonia	22 (12.6)	14 (8.0)	8 (4.6)
Dyspnea	7 (4.0)	7 (4.0)	0
Cough	63 (36.2)	63 (36.2)	0
Hemoptysis	6 (3.4)	6 (3.4)	0
Arrhythmias	8 (4.6)	8 (4.6)	0
Chest discomfort	22 (12.6)	22 (12.6)	0
Myocarditis	5 (2.9)	4 (2.3)	1 (0.6)
Elevated liver enzymes	19 (10.9)	17 (9.8)	2 (1.1)
Hypothyroidism	28 (16.1)	28 (16.1)	0
Hyperthyroidism	13 (7.5)	13 (7.5)	0
Anemia	67 (38.5)	54 (31.0)	13 (7.5)
Leukopenia	94 (54.0)	60 (34.5)	34 (19.5)
Neutrophilic granulopenia	90 (51.7)	50 (28.7)	40 (23.0)
Thrombocytopenia	46 (26.4)	40 (23.0)	6 (3.4)
Fatigue	70 (40.2)	66 (37.9)	4 (2.3)
Edema	11 (6.3)	11 (6.3)	0
Anorexia	47 (27.0)	47 (27.0)	0
Hypoalbuminemia	33 (19.0)	33 (19.0)	0
Nausea	57 (37.6)	57 (37.6)	0
Vomiting	51 (29.3)	50 (28.7)	1 (0.6)
Diarrhea	39 (22.4)	37 (21.3)	2 (1.1)
Constipation	18 (10.3)	18 (10.3)	0
Electrolyte disturbance	22 (12.6)	22 (12.6)	0
Radiation esophagitis	9 (5.2)	9 (5.2)	0
Sore throat	13 (7.5)	13 (7.5)	0
Elevated lactate dehydrogenase	23 (13.2)	23 (13.2)	0
Rash	7 (4.0)	7 (4.0)	0
Cutaneous capillary hemangioma	95 (54.6)	94 (54.0)	1 (0.6)
Myelitis	1 (0.6)	0	1 (0.6)
Peripheral sensory neuropathy	2 (1.1)	2 (1.1)	0

## Discussion

In our real-world study, we used real-world data to analyze the efficacy and safety of monotherapy and combination therapy with camrelizumab in clinical practice in China, and the results showed that (1) camrelizumab monotherapy and combination therapy displayed satisfactory DCRs, and the DRCs were all higher than 90%; (2) receiving > 4 cycles of camrelizumab had a better 2-year OS (33.4% vs. 30.9%) than receiving ≤ 4 cycles of camrelizumab; (3) the median PFS duration of the whole cohort was 8 months, and to our knowledge, the result was better than that reported in the previous studies; (4) there was a significant association between LIPI and survival for patients treated with camrelizumab + radiotherapy, which leads to a hypothesis that LIPI may be a crucial indicator showing which patients are likely to repose well to camrelizumab + radiotherapy; and (5) camrelizumab showed manageable treatment-related AEs, and the most common immune-related AE was immune-related reactive capillary endothelial proliferation. Therefore, it can be considered a safe and efficient treatment method.

Before 2019, a combination of 5-fluoropyrimidine and platinum-based chemotherapy was widely accepted worldwide as the first-line therapy for treating unresectable advanced and metastatic EC cases. Various chemotherapy strategies showed a median OS of ~ 10 months for advanced and metastatic EC [18, 19]. In the event of first-line therapy failure, the subsequent second-line treatment options are limited, further leading to a dismal survival benefit. PD-1 and PD-L1 is a molecule pair that performs T cell inhibitory functions [20]. The PD-L1 expression was detected on tumor surface, and it binds with PD-1, which is expressed on the T cell surface. Immune checkpoint inhibitors (ICIs) based on the PD-1 pathway blockade have been proven to cause tumor cell regression [21]. Several clinical studies have demonstrated promising survival benefit and manageable safety profile of ICIs in several cancer types, including non-small cell carcinoma, head and neck cancer, and ESCC [22, 23]. The phase 3 KEYNOTE-181 was a randomized study of second-line pembrolizumab monotherapy versus investigator’s choice chemotherapy in EC patients with advanced/metastatic EC; in this study, compared with investigator’s choice chemotherapy, pembrolizumab showed a significantly increase in OS of patients with a PD-L1 CPS of ≥ 10 (9.3 months vs. 6.7 months, *P* = 0.0074). Fewer patients had treatment-related AEs (any grade) in the pembrolizumab group than in the chemotherapy group [12]. The phase 3 ATTRACTION-3 was a randomized multicenter study of second-line nivolumab versus chemotherapy in patients with unresectable advanced or recurrent ESCC. Regardless of their PD-L1 status, 419 patients were enrolled. Nivolumab was superior to chemotherapy in terms of OS (10.9 months vs. 8.4 months, *P* = 0.019). Grade 3 or 4 treatment-related AEs were found in 18% patients in the nivolumab group versus 63% patients in the chemotherapy group [11]. Based on these results, nivolumab and pembrolizumab were approved by the United Stated Food and Drug Administration as second-line ICI treatments of ESCC. In 2021, KEYNOTE-590, a randomized, placebo-controlled, phase 3 study, revealed that pembrolizumab + chemotherapy was superior to placebo + chemotherapy in terms of the OS in all randomized patients regardless of their CPS (12.4 months vs. 9.8 months, *P* < 0.001) and in patients with ESCC (*P* = 0.0006), PD-L1CPS ≥ 10 (*P* < 0.0001), and ESCC PD-L1 ≥ 10 (*P* < 0.0001) [13]. Therefore, pembrolizumab was first approved for locally advanced or metastatic EC regardless of histology and PD-L1 CPS status. Several randomized phase 3 studies that followed, such as ESCORT and ESCORT-1st, also reported better safety and efficacy of ICIs in patients with EC. ESCORT and ESCORT-1st showed that camrelizumab monotherapy and camrelizumab combined with chemotherapy both significantly improved the OS as second- and first-line therapies in Chinese patients with advanced or metastatic ESCC, respectively. Therefore, Chinese National Medical Products Administration has approved camrelizumab monotherapy as the second-line treatment and camrelizumab + chemotherapy as the first-line treatment for treating ESCC regardless of the PD-L1 CPS status. The RCTs mentioned above have proven the efficacy of ICIs, including camrelizumab, in locally advanced ESCC. These results were consistent with those of our present study. Our results showed that the median PFS was 7 months and 11 months in patients who received camrelizumab as the second- and first-line therapy, respectively. The median OS was 11 months and 16 months in patients who received camrelizumab as the second- and first-line therapy, respectively. Moreover, the DCR of patients in the camrelizumab monotherapy group, camrelizumab + CT group, and camrelizumab + RT group was 92.3%, 90.6%, and 96.1%, respectively. Our real-world data demonstrate that camrelizumab is a safe and efficient treatment option for patients with uncontrolled ESCC. However, except for the expression of PD-L1 expression, no other specific biomarkers that reflect the clinical efficacy of immunotherapy for ESCC are known. Thus, it is necessary to explore other important prognostic indicators to predict which ESCC patients can achieve survival benefit from camrelizumab.

Several studies have demonstrated that systemic inflammatory response significantly boosts cancer cell growth, primary tumor invasion, distant metastasis, and immune tolerance [24–27]. Tumor cells obtain energy from aerobic glycolysis. Cancer cells promote the expression of aerobic glycolysis enzymes to maintain cancer cell growth. Lactate dehydrogenase (LDH) is a crucial glycolysis enzyme [28–30]. Inflammatory-based prognostic factors, such as neutrophil-to-lymphocyte ratio (NLR) and serum LDH, are associated with a poor prognosis and have shown potential value to predict prognosis in patients with several cancer types [31–35]. However, whether pretreatment inflammatory marker and serum LDH levels can be treated as predictors of benefits from ICIs remains unclear. In 2018, Mezquita et al. studied 466 patients with advanced non-small cell lung cancer receiving PD-1/PD-L1 inhibitors and were the first to develop the LIPI to investigate the relationship between poor outcomes of PD-1/PD-L1 inhibitors and the LIPI score. In that study, the LIPI score was defined on the basis of NLR greater than 3 and LDH greater than the upper limit of the normal value and divided into three groups (good, 0 factors; intermediate, 1 factor; and poor, 2 factors). The median OS was 3 months, 10 months, and 34 months for poor, intermediate, and good LIPI groups, respectively (*P* < 0.001). Moreover, the median PFS was 1 month, 3 months, and 6 months for poor, intermediate, and good LIPI groups, respectively (*P* = 0.001). LIPI can predict ICIs’ treatment outcomes and can be a useful indicator of NSCLC patients likely to benefit from ICIs [36]. In 2019, Sorich et al. studied 1489 NSCLC patients treated with ICIs and found that the median PFS ranged from 1.4 months for the poor LIPI group to 4.2 months for the good LIPI group. In addition, the median OS ranged from 4.5 months for the poor LIPI group to 18.4 months for the good LIPI group. Good LIPI was associated with significantly prolonged OS (*P* < 0.001) and PFS (*P* < 0.001) in NSCLC patients treated with ICIs. Moreover, in chemotherapy-treated NSCLC patients, pretreatment LIPI was also statistically significantly associated with OS and PFS (*P* < 0.001). LIPI can also be viewed as a potential prognostic indictor for survival of NSCLC patients treated with chemotherapy. Notably, LIPI has also been investigated in patients with extrapulmonary cancers [37]. Feng et al. studied 361 ESCC patients who underwent curative esophagectomy and found that the 5-year cancer-specific survival rates associated with LIPI 0, LIPI 1, and LIPI 2 were 40.9%, 19.0%, and 9.8%, respectively. Multivariate analysis also revealed that LIPI was an independent predictor of cancer-specific survival in patients with resected ESCC [38]. In our opinion, LIPI could enlarge the scope of the role in predicting the prognosis of different treatment methods and malignant tumors. In the present study, we found good LIPI to be positively associated with prolonged PFS and OS in patients with ESCC who received camrelizumab. Consistently, multivariate analyses also showed that good LIPI independently predicted better PFS and OS. Consequently, LIPI may be an efficient predictor of the efficacy of camrelizumab therapy. Then, we performed further exploratory subgroup analyses and found that compared to patients with good LIPI, those with intermediate-to-poor LIPI showed a benefit in the camrelizumab + RT group; however, the benefit was not statistically significant in terms of their PFS (*P* = 0.074) and OS (*P* = 0.0076).

To our knowledge, this is the first multicenter real-world study of its kind to (i) evaluate the efficacy and safety of camrelizumab in patients with unresectable advanced, recurrent, or metastatic ESCC, (ii) assess the predictive value of LIPI to justify the addition of camrelizumab to radiotherapy, and (iii) identify high-risk patients who could benefit from camrelizumab monotherapy and combination treatment. Based on the results of our real-world study, camrelizumab monotherapy and combination therapy can be considered as safe and efficient treatment methods for unresectable advanced, recurrent, or metastatic ESCC. Moreover, high-risk ESCC patients with intermediate-to-poor LIPI are not able to achieve satisfactory tumor response and survival benefits from camrelizumab monotherapy or combination therapy. The findings of our study suggest two specific areas for future studies to focus on, which are exploring the underlying mechanisms associating LIPI with camrelizumab response and identifying other biomarkers that can predict the treatment response of ICIs. In the future, phase 3 randomized clinical trials with diverse populations are needed to validate our findings.

This study has some limitations. First, the study population was relatively small. Second, the enrolled patients treated with camrelizumab monotherapy and camrelizumab + RT increase bias in this study. In the real world, a large proportion of ESCC patients is the elderly with severe complications. These patients could not accept the standard concurrent camrelizumab + CT. Camrelizumab monotherapy and camrelizumab + RT were still used in clinical practice. Third, we only analyzed ESCC patients in this study. Whether the results can be applied to esophageal adenocarcinoma still needs further investigation. Fourth, we did not analyze the impact of the PD-L1 expression in most patients. The association of the PD-L1 expression status and efficacy of camrelizumab remains unclear. Large-scale, multicenter, randomized studies with consistent study procedures, strict inclusion criteria, and diverse populations are warranted to mitigate the potential biases and limitations of the current study and further validate our findings.

## Conclusion

Our study showed that camrelizumab as both monotherapy and combination therapy was safe and effective for patients with unresectable advanced, recurrent, or metastatic ESCC. Receiving > 4 cycles of camrelizumab and having a good LIPI were correlated with a longer OS and PFS. Patients with a good LIPI benefited from a combination of camrelizumab and local RT. We believe that LIPI can serve as a prognostic biomarker for ESCC patients receiving camrelizumab + RT.

## Data Availability

The data sets used and /or analyzed during the current study are available from the corresponding author on reasonable request.
